# The Role of Genetic Testing in the Evaluation of Dilated Cardiomyopathies

**DOI:** 10.1155/2021/6641108

**Published:** 2021-03-02

**Authors:** Kolade M. Agboola, Trevon McGill, Barry A. Boilson, Naveen L. Pereira, Jacob C. Jentzer

**Affiliations:** ^1^Department of Cardiovascular Medicine, Mayo Clinic College of Medicine, Rochester, MN 55905, USA; ^2^Department of General Internal Medicine, Mayo Clinic College of Medicine, Rochester, MN 55905, USA

## Abstract

We present an adolescent African American male admitted to the cardiac intensive care unit with cardiogenic shock and acute respiratory failure. Through an overview of his presentation, diagnostic workup, and treatment, we demonstrate the clinical utility of genetic testing in the evaluation of unexplained dilated cardiomyopathies.

## 1. History of Presentation

Mr. A is a 19-year-old adopted African American male who presented to our cardiac intensive care unit with cardiogenic shock and acute respiratory failure requiring intubation.

The patient initially presented to an outside hospital with a several week history of chest pain and progressive shortness of breath and was hospitalized for 10 days prior to transfer. He was started on noninvasive positive-pressure ventilation for hypoxemic respiratory failure and severe acute respiratory syndrome. Coronavirus-2 (SARS-CoV-2) PCR testing was negative. He did not tolerate noninvasive ventilation, necessitating intubation and mechanical ventilation. Following intubation, he developed profound bradycardia and suffered a brief pulseless electrical activity cardiac arrest with return of spontaneous circulation after two minutes of cardiopulmonary resuscitation.

The patient was transferred to our cardiac intensive care unit while he was intubated, sedated, and supported on vasopressors. The history of present illness was provided by the patient's mother, who reported that the patient was previously relatively active and enjoyed playing sports. However, over the past few months, the patient had asked his mother for rides to the basketball court when he previously walked, and he had been bringing a chair to sit and rest on when he plays. He did not report any overt signs of shortness of breath, palpitations, or chest pain.

Initial physical examination revealed a well-developed, well-nourished young African American male without syndromic features. He was sedated with an endotracheal tube in place. Breath sounds were coarse bilaterally. Cardiovascular examination demonstrates a regular tachycardia, normal first and second heart sounds, and no appreciable murmurs or gallop. His extremities were cool to touch, and he had mild pretibial pitting edema bilaterally.

## 2. Past Medical History

Medical history was significant for developmental delay of unknown etiology, major depressive disorder, and anxiety. Cardiovascular risk factors include diabetes mellitus type 2 and morbid obesity. Family history was not available due to the patient's adopted status.

## 3. Differential Diagnosis

The range of possible etiologies for a young adult with cardiogenic shock and acute respiratory failure includes pulmonary embolism, acute fulminant myocarditis, acute myocardial infarction triggered by drug use or spontaneous coronary dissection, and acute decompensated heart failure from cardiomyopathy.

## 4. Investigations

**Figure 1:**
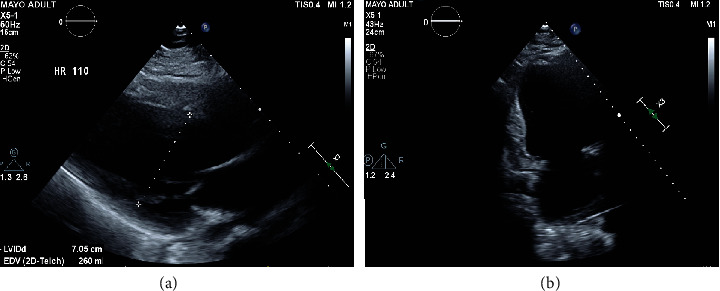
Transthoracic echocardiogram images in the (a) parasternal long axis and (b) apical 2-chamber views demonstrating profound left ventricular dilatation, as well as left atrial enlargement.

**Figure 2:**
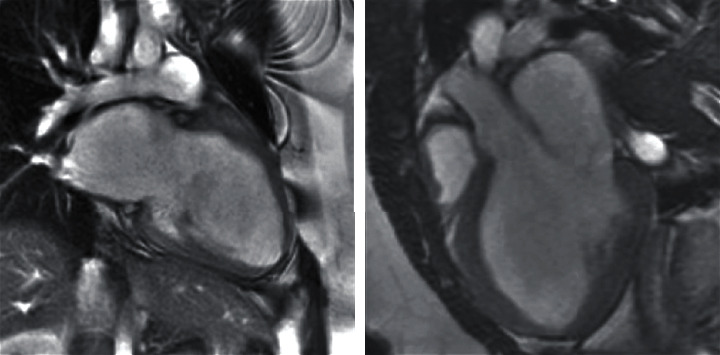
Cardiac magnetic resonance imaging video clips depicting severe left ventricular enlargement and markedly reduced left ventricular systolic function.

Transthoracic echocardiogram revealed a severely enlarged left ventricle with biplane ejection fraction of 20% and severe global hypokinesis (Figure 1). There was mild right ventricular chamber enlargement with mildly decreased systolic function. Cardiac troponin levels were mildly elevated, and urine drug screen was negative. The patient underwent right heart catheterization with endomyocardial biopsy. Pathology did not show evidence of myocarditis or active inflammation. Cardiac magnetic resonance imaging demonstrated a severely enlarged left ventricular chamber size with left ventricular ejection fraction 32% and global hypokinesis (Figure 2). No myocardial edema was appreciated and delayed postcontrast images revealed no late gadolinium enhancement. Viral screening for enterovirus, parvovirus B19, varicella-zoster, cytomegalovirus, herpes simplex virus-1, herpes simplex virus-2, and SARS-CoV-2 was performed, all of which were negative. Genetic testing was offered given the patient's extremely young age and the severe degree of systolic dysfunction. Sequencing using a cardiomyopathy-specific gene panel, which tests for variants within a total of 67 genes, demonstrated heterozygosity for a rare variant that was likely pathogenic involving the RBM20 gene, which encodes a splicing factor that targets multiple cardiac genes including titin. The specific variant is a single nucleotide polymorphism which replaces a cytosine nucleotide with a thymine nucleotide, resulting in a premature translational stop codon and absent or markedly disrupted protein production.

## 5. Management

The patient's cardiogenic shock and decompensated heart failure were managed with aggressive diuresis and intermittent inotropic support. The patient developed recurrent atrial arrhythmias and ultimately underwent atrial flutter ablation. He did not have a significant ventricular arrhythmia burden during his hospitalization. Once he was hemodynamically stable without inotropic support, guideline-directed medical therapy with a beta blocker, angiotensin receptor-neprilysin inhibitor, and aldosterone antagonist was initiated. Respiratory recovery was hindered by critical illness myopathy and prolonged intubation requiring tracheostomy placement to liberate from the ventilator.

## 6. Discussion

Dilated cardiomyopathy (DCM) is the second most common etiology of heart failure with reduced ejection fraction after ischemic cardiomyopathy, with an estimated prevalence of 30% to 40% of cardiomyopathies [1]. Approximately 30% to 50% of DCMs are due to familial cardiomyopathies with an identifiable genetic cause found in up to 40% of these, and genetically determined DCM is perhaps the single most common cause of DCM [1]. However, our understanding of the underlying genetic mechanisms of DCM remains poorly understood and underexplored. Although there is now great ability to test for genetic variants with whole exome or genome sequencing, the clinical significance of findings and associated phenotypes is often unknown.

RNA binding motif 20 (RBM20) is a splicing factor responsible for alternative splicing of several proteins expressed within cardiomyocytes, including the sarcomeric protein titin [2]. Truncating variations in the gene encoding titin are among the most common genetic causes of DCM and can be seen in 10 to 20 percent of patients with DCM [3]. Additionally, RBM20 controls splicing of many proteins involved in intracellular calcium handling, thereby leading to a propensity for ventricular arrhythmia in the setting of abnormal RBM20 function [4]. There are specific exons in which genetic variation has been demonstrated to be particularly deleterious. For example, transcription errors in exons 9 and 10 are associated with high penetrance of cardiomyopathy with a high arrhythmia burden, including atrial and ventricular tachyarrhythmias [4]. Animal models with RBM20 knockout mice have demonstrated increased intracellular calcium levels and increased sarcoplasmic reticulum calcium content compared to wild-type controls; these findings are also seen with heterozygous loss of RBM20 [5].

A similar phenotype is observed among humans with RBM20 genetic variation. A database study of DCM patients reveals a relatively younger age at diagnosis among individuals with a RBM20 variant. Additionally, patients with RBM20-associated cardiomyopathy are more likely to endorse a family history of DCM, suggesting a high degree of penetrance associated with this specific variant. Of note, a family history of sudden cardiac death was reported in 51% of RBM20 registry patients [4]. An increased burden of arrhythmia including atrial fibrillation, nonsustained ventricular tachycardia, and ventricular tachycardia necessitating implantable cardioverter-defibrillator (ICD) placement has been described among RBM20 DCM patients [4].

Interestingly, we present a patient ultimately found to have a genetic variant involving exon 2 of RBM20 who presents with an aggressive DCM accompanied by significant atrial arrhythmias, requiring catheter ablation. Pathogenic variations in exons 6-9 of RBM20 have previously been identified in individuals with DCM [6]. However, to the best of our knowledge, variants in the exon 2 gene have not previously been characterized as penetrant with regard to DCM and arrhythmia.

## 7. Conclusion

We present the case of an adolescent male presenting with new acute decompensated DCM resulting in cardiogenic shock. With rapid advances in genetic medicine and the availability of genetic diagnostics, providers can more readily integrate genetic testing into clinical practice. This case demonstrates the utility of genetic testing in the evaluation and treatment of DCM. With the use of such advances, we are now able to better characterize and understand the underlying genetic mechanisms of heart failure thus offering a molecular diagnosis to patients with DCM. Furthermore, understanding the role of genetics in DCM will ultimately allow for enhanced familial counseling, prognostication, and treatment for patients in addition to providing insights into normal cardiac function [7]. Identification of potentially arrhythmogenic genetic variants associated with DCM may facilitate decision-making regarding ICD implantation or the use of a wearable defibrillator in the early postdiagnosis period. As in our case due to the high arrhythmia risk conferred by his specific genetic variant, the patient was outfitted with a LifeVest wearable defibrillator prior to discharge. Although this practice is not recommended in the management guidelines for patients with RMB20 variants, we felt it was reasonable to recommend as the patient's true arrhythmia burden was unknown at the time of discharge. Future research will be needed to better characterize the divergent natural history of genetically determined DCM as a function of the affected gene and specific mutation, to help clinicians predict which patients may develop progressive or end-stage heart failure requiring early consideration for transplant.

## 8. Learning Objectives

1. To understand the significance of genetics in idiopathic nonischemic cardiomyopathy
2. To investigate the implication of RNA binding motif 20 variations in the pathogenesis of dilated cardiomyopathy
3. To demonstrate the benefit and utility of genetic testing in the evaluation and treatment of individuals with idiopathic dilated cardiomyopathy

## Additional Points

*Tweet*. Check out our case report demonstrating the utility of genetic testing in individuals with dilated cardiomyopathies and its implications on patient management.
